# Urban–rural social security, adult children’s layoffs and older parents’ health

**DOI:** 10.3389/fpubh.2025.1619806

**Published:** 2025-09-03

**Authors:** Yifei Yang

**Affiliations:** School of Public Administration, Shanghai University of Finance and Economics, Shanghai, China

**Keywords:** mass layoffs, health of older adults, urban–rural disparities in social security, intergenerational support, machine learning

## Abstract

**Objective:**

Aging population and rising unemployment risks have emerged as dual challenges for governments worldwide. Using China’s 1990s state-owned enterprise layoffs as a natural experiment, we examine causal evidence on how adult children’s unemployment affects older parents’ health. We particularly analyze how urban–rural social security moderates these effects.

**Methods:**

Using data from the China Health and Nutrition Survey 1991–2006, we construct three health outcome dimensions for older parents: disease status, daily functional abilities (physical function and instrumental activities of daily living), and body mass index. Econometric analysis uses a two-way fixed effects model and a causal forest model.

**Results:**

The layoffs of adult children significantly increased older parents’ morbidity risk. Due to disparities in the social security system between urban and rural household registration (hukou), this effect primarily increased health risks—including higher probability of disease, increased risk of hypertension, limitations in daily activities, and underweight conditions—among rural-hukou older parents living in urban areas. In contrast, layoffs of adult children showed no measurable impact on urban-hukou older parents. Mechanistically, layoffs did not reduce daily care provided by children to their parents, but rather diminished household income. Moreover, layoffs of adult children reduced older parents’ healthcare utilization and lowered their nutritional intake. Rural-hukou older parents living in urban areas were most affected, with reduced protein and fat consumption.

**Conclusion:**

The reduction in family resources caused by adult children’s unemployment is detrimental to older parents’ health. The social security system serves as a vital safety net for protecting the health of older populations. Strengthening social security is an essential policy complement to mitigate welfare losses in families affected by unemployment.

## 1 Introduction

Amid rapid technological disruption and uncertainty in trade policies, the risk of structural unemployment and large-scale job losses has become a growing concern. According to the World Economic Forum’s 2025 Employment Report, 22 percent of jobs worldwide will be transformed or displaced by 2030, with 170 million new positions emerging alongside the displacement of 92 million.^1^ Geo-economic tensions and protectionist policies further amplify the risk of large-scale unemployment. In China, the urban surveyed unemployment rate is anticipated to reach 5.5 percent in 2025.^2^ Unemployment remains a critical issue in health economics research. Unemployed individuals face severe economic deprivation, compromising households’ ability to meet basic subsistence needs. Existing studies have shown that unemployment is detrimental to individual health, children’s health, and broader social stability (1–3). Furthermore, unemployment exerts multigenerational impacts—not only affecting individuals and their offspring, but also influencing older parents’ health through upward intergenerational support mechanisms, an underexplored dimension in the literature.

The intersecting challenges of population aging and rising unemployment make it essential to investigate how adult children’s job loss affects older parents’ health through household-level analysis.

By the end of 2024, individuals aged 60 and above accounted for 22.0 percent of China’s total population.^3^ According to the Seventh National Population Census, 12.7 percent of older adults reported poor health status, and nearly 600,000 experienced disabilities in activities of daily living. These statistics highlight older adults’ health as an urgent social concern at the crossroads of healthcare and older adult care systems. China has a long-standing tradition of family-based older adult care. As of 2020, 33 percent of older individuals relied mainly on financial support from family members.^4^ In this context, adult children’s economic and caregiving contributions play a crucial role in shaping older adults’ health outcomes (4). Disruptive life events such as unemployment may reduce intergenerational transfers or even reverse them—from older parents to their adult children—thus undermining parental well-being (5). However, due to the complex endogenous relationship between adult children’s unemployment and older parents’ health, addressing potential confounding factors remains a core challenge in empirical research.

The reform of state-owned enterprises in China in the 1990s and the resulting mass layoffs provide an ideal quasi-natural experiment to examine how adult children’s unemployment affects older parents’ health. Unlike in developed economies, the state-owned enterprise (SOE) reforms of the 1990s were implemented under a system of *work-unit socialism* and a limited social security infrastructure, making it difficult for laid-off workers and their families to access benefits such as unemployment insurance. Meanwhile, the underdeveloped private sector in China at the time was unable to absorb the surge in laid-off workers, resulting in a prolonged period of labor market distress. Prior research has primarily examined the impact of layoffs on downward intergenerational transfers—for instance, showing that the children of laid-off workers receive reduced investment in human capital (6). In contrast, this study shifts the focus to upward intergenerational support, investigating whether layoffs impair adult children’s ability to support their older parents, thereby compromising older parents’ health outcomes.

The social security system in China offers basic livelihood protection and employment assistance to unemployed individuals and their families. However, persistent disparities in the allocation of social security resources between urban and rural areas remain a major concern. China’s urban and rural household registration (hukou) system institutionalizes unequal access to social welfare benefits based on hukou registration (7). Particularly before the implementation of the New Rural Pension Scheme and the New Rural Cooperative Medical Scheme, individuals with rural hukou faced severe deficiencies in basic social security coverage. The difference in the level of social old-age care for the older adults between urban and rural areas is substantial. Because formal social insurance can substitute for family-based older adult care, it reduces older adults’ reliance on their children for both financial support and daily assistance (8). Consequently, when family support capacity is weakened, rural-hukou older adults—who disproportionately lack social security coverage—are more vulnerable to shortfalls in healthcare access and retirement security.

Leveraging the large-scale layoffs triggered by China’s SOE reforms in the 1990s as a quasi-natural experiment, this study examines how adult children’s job loss affects the health of their older parents, with particular attention to the heterogeneous impacts arising from disparities in urban–rural social security systems. Using panel data from the China Health and Nutrition Survey (CHNS, 1991–2006), we apply two-way fixed effects models and causal forest analysis to identify both average and heterogeneous treatment effects. Our findings show that adult children’s layoffs significantly deteriorate older parents’ health, especially among rural-hukou parents living in urban areas. This study contributes to a broader understanding of the social consequences of labor market shocks in developing and transitional economies.

## 2 Institutional context: SOE layoffs wave

The mass layoffs resulting from China’s SOE reforms are so designated due to their unprecedented scale and profound societal impact. During the 1990s, SOEs faced mounting challenges following China’s market-oriented reforms, compelling many inefficient state-owned firms to implement necessary restructuring measures, including bankruptcies, dissolutions, and reorganizations. While these workforce reductions successfully alleviated operational deficits and significantly boosted macroeconomic efficiency (9), they simultaneously triggered historically large-scale worker layoffs across state industries (10).

Employment in China’s state-owned sector declined sharply from 106.6 million workers in 1991 to 64.31 million in 2006, representing a reduction of approximately 40 million positions.^5^ From 1990 to 1997, state enterprises had already laid off 15 million employees. In 1998, the central government announced a three-year plan to revitalize financially distressed SOEs through institutional reform, organizational restructuring, and management enhancement.^6^ Official statistics from the National Economic and Social Development Statistical Bulletins (1998–2000) show that annual new layoffs fell from 5.62 million to 4.21 million during this period.^7^ The downward trend continued through 2000–2004, with both new layoffs declining and reemployment rates improving. By the end of 2005, the total stock of laid-off SOE workers had plummeted to 610,000 from its peak of over 6.5 million (11).

Workers laid off during the reform of SOEs suffered substantial and persistent income losses (12). At the time, China’s market economy remained underdeveloped. Most laid-off workers faced serious reemployment challenges due to information asymmetries and limited job opportunities, leading to abrupt income losses. Many displaced workers were forced into low-skilled jobs or informal employment arrangements, where their productive contributions were inadequately compensated. Without adequate social protection systems, such as unemployment insurance, most laid-off workers struggled to escape poverty.

The mass layoffs during SOE reforms significantly eroded workers’ welfare: they experienced worsened health outcomes (13), while reduced household resources negatively impacted children’s health and educational attainment (6, 14). Building on this evidence, our study examines how layoffs affect upward intergenerational support within families. By treating these policy-induced mass layoffs as an exogenous shock to adult children’s employment status, we establish a cleaner identification strategy to assess the causal impact of children’s unemployment on older parents’ health.

## 3 Theoretical framework

### 3.1 The impact of adult children’s intergenerational support on parental health

Population aging has placed substantial pressure on healthcare and older adult care systems. Under China’s family-based older adult care model, children’s support behaviors directly impact parental health (15). Adult children’s unemployment, for example, may disrupt intergenerational transfers.

Intergenerational support from adult children effectively improves the health outcomes of older parents. Key family support resources (such as household size, living arrangements, and children’s status) significantly influence parental health (16). There are two main types of intergenerational support: financial support and daily care. Financial support from adult children is beneficial to parental health, as evidenced by older adult recipients of substantial transfers spending more on medical care (17). Daily care provided by adult children also has a significant positive impact on the survival and health status of older adults. For instance, older adults who receive more instrumental support exhibit a lower incidence of catastrophic medical expenditures (18).

When adult children’s support capacity is negatively affected by shocks such as unemployment, their parents’ health status tends to deteriorate. Adult children’s economic and social standing constitutes a critical determinant of older parents’ health outcomes (19). Empirical studies demonstrate that unemployed children typically reduce intergenerational support to parents, sometimes even triggering reverse financial transfers (from older parents to adult children) (20). Labor market shocks to adult children can reduce older parents’ retirement savings, food consumption, and increase labor supply and demand for social security among the non-retired older population (21). Adult children experiencing unemployment, divorce, or health problems provide significantly less daily support, ultimately worsening parental welfare (22).

Under China’s tradition of filial piety-based care for older adults, children serve as an important safeguard for their older parents. However, existing studies often examine this issue in the context of more comprehensive unemployment protection, making it difficult to disentangle the role of unemployment insurance and address the endogeneity between children’s job loss and parental health. The mass layoffs during China’s SOE reforms offer a unique natural experiment to study this dynamic. Leveraging this exogenous policy shock, our research examines how downward labor market shocks affect upward support flows within families, thereby contributing to the literature on older adults’ health.

### 3.2 The impact of social security on older adults’ health

Social security serves as a crucial supplement to family-based care for older adults and plays a significant role in enhancing older adults’ health. However, the urban–rural disparity in China’s social security system has resulted in varying levels of social protection for older populations with different hukou statuses, leading to significant health inequalities. During the study period (late 20th to early 21st century), rural older adults with agricultural hukou faced a severe lack of social security. Unlike their urban counterparts, they were critically dependent on support from their children.

China’s social security resource allocation has long exhibited urban–rural disparities. Initially, the system was not universal but operated through work units (danwei) (23). Regarding social insurance, the basic old-age insurance initially covered only urban enterprises and their employees. Rural residents were excluded until the 2009 nationwide pilot of the New Rural Pension Scheme.^8^ The New Rural Cooperative Medical Scheme (NRCMS) initially provided minimal protection, as it failed to significantly reduce out-of-pocket medical expenses or improve health outcomes for rural residents. It was not formally established as the basic medical insurance system for rural areas until 2009 (7). Even in social assistance programs, urban minimum living standards have consistently exceeded rural standards nationwide. Therefore, during the mass layoff period, lacking urban hukou meant a lack of effective social security protection.

Disparities in social security coverage result in urban and rural older adults differing in their dependence on familial support. Social insurance can reduce family caregiving time and intergenerational financial support, decreasing older parents’ reliance on adult children while not negatively impacting their physical and mental health (24). Consequently, urban–rural disparities in social security contribute to health inequalities among older populations with different hukou statuses. Existing studies have found that social insurance can improve healthcare utilization among insured older adults, but urban employee medical insurance has a greater effect in this regard than the NRCMS for rural residents (25). This implies that during the mass layoff period, rural-hukou older adults were more dependent on their adult children for support than urban-hukou older adults.

Existing literature highlights social security’s complementary role in supporting adult children’s contributions and notes urban–rural disparities in coverage. Building upon this foundation, this paper further examines the safety-net function of social security when adult children’s support capacity is insufficient, offering theoretical support for promoting urban–rural social security integration.

### 3.3 Urban–rural social security, adult children’s layoffs and older parents’ health

As a form of involuntary unemployment, layoffs caused substantial welfare losses for affected workers. During the late 20th century, China’s underdeveloped market economy made reemployment extremely difficult for laid-off workers, resulting in substantial household income reductions. The mandatory termination of employment relationships without adequate unemployment insurance coverage fundamentally compromised these families’ basic living standards. Within China’s traditional “raising children for old-age support” framework, older adults primarily relied on family-based care due to two key factors: First, the inadequate social security system failed to provide viable alternatives to family support. Second, their limited personal financial capacity necessitated dependence on younger generations. When adult children’s ability to provide financial support diminished due to job displacement, older parents suffered health deterioration. Based on this mechanism, we propose the following hypothesis:

*H*1: Employee layoffs resulting from SOE reforms adversely affect their parents’ health.

Social security reduces older parents’ reliance on adult children for financial support and daily care, supplementing the family-based older adult care system. However, during the wave of mass layoffs, China’s social security system was characterized by a severe urban–rural segmentation (26). Rural-hukou holders lacked access to adequate social protection. Many rural-hukou migrants in China did not successfully transfer their household registration when moving to cities. This created a mismatch between individuals’ place of residence and their registered hukou location (27). For example, some older parents moved to cities with their adult children but retained their rural hukou status. Rural-hukou older adults living in urban areas often lacked access to the same social security benefits as their urban-hukou counterparts. As a result, rural-hukou older parents were more dependent on their adult children’s support than urban-hukou older parents. The unemployment of adult children undermined the family’s capacity to provide care for older adults. Urban-hukou older parents, with more comprehensive social security coverage, were better protected from the adverse effects of weakened intergenerational support. In contrast, rural-hukou older parents lacking social security protection suffered more severe negative effects from such disruptions. Based on urban–rural disparities in social security, we propose the second theoretical hypothesis:

*H*2: Among older parents living in urban areas, those with rural hukou status experience greater health deterioration following their adult children’s layoffs than older parents with urban hukou status.

Financial support is the primary form of intergenerational transfers from adult children to their parents, and layoffs undermine this by reducing workers’ disposable income. Extensive research has documented the positive health effects of economic support through two key mechanisms: higher disposable income facilitates better nutritional intake among the older adults (28), and it enhances healthcare utilization, thereby improving health outcomes. The SOE reforms and resulting involuntary job losses reduced household incomes, diminishing the economic support available to older parents. This was reflected in reduced nutritional intake and lower healthcare utilization—both of which ultimately compromised the health of older parents. Given the urban–rural disparities in social security during this period, these negative effects were likely amplified for rural-hukou older adults living in urban areas. In contrast, the job displacement may have increased adult children’s available time, potentially allowing them to provide more daily care, which could have beneficial effects on parental health. Integrating these pathways, we propose our third hypothesis:

*H*3: Employee layoffs reduce household income, leading to reduced nutritional intake and healthcare utilization among older parents, ultimately impairing their health outcomes.

**Figure 1:**
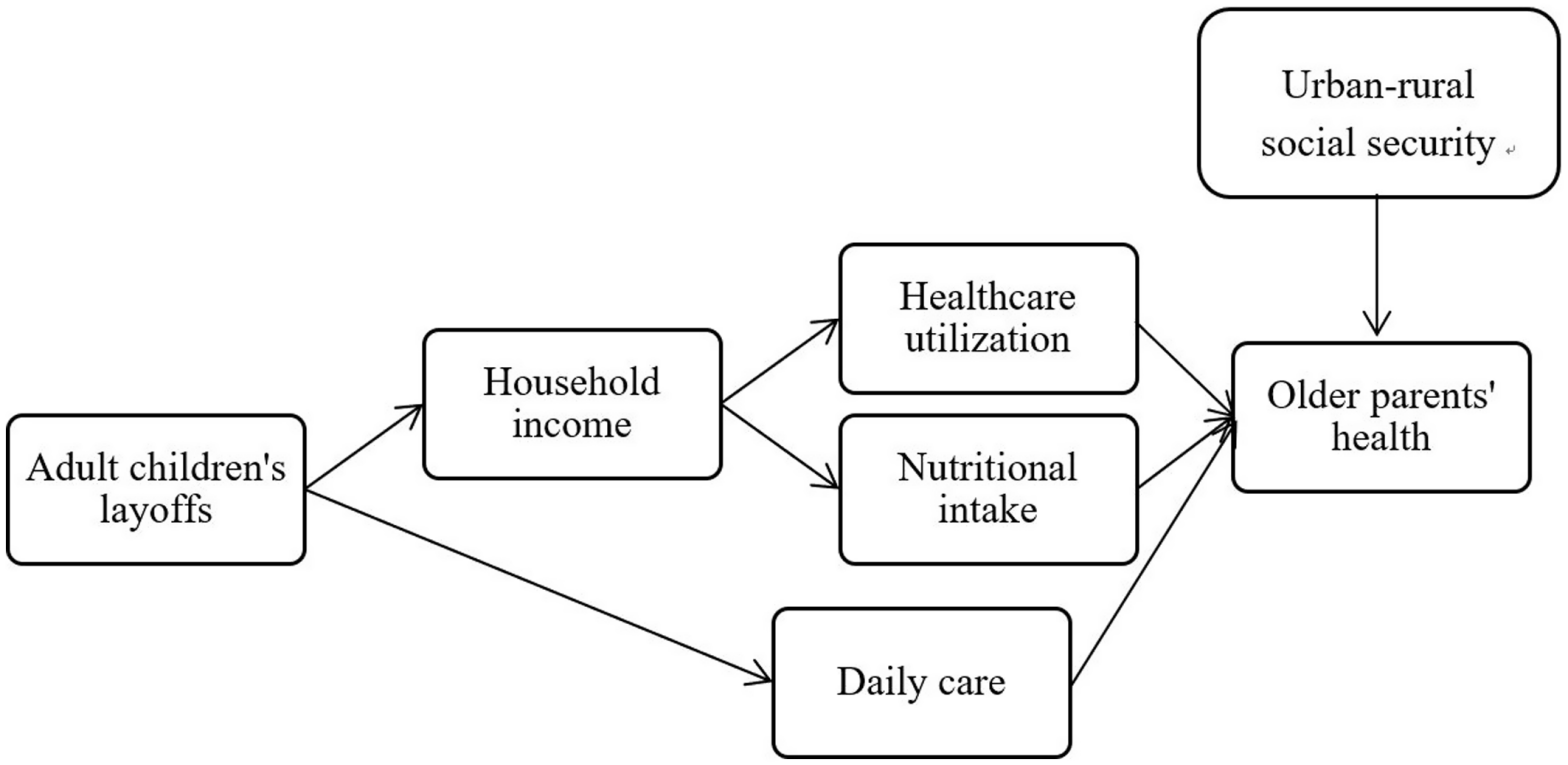
Mechanism pathway.

Based on the above theoretical analysis, the study’s conceptual framework is presented in Figure 1.

## 4 Research methods

### 4.1 Data sources

This study uses household-level longitudinal survey data from the CHNS, an international collaborative project between the Carolina Population Center at the University of North Carolina and the Chinese Center for Disease Control and Prevention. The dataset covers 15 provinces and municipalities with significant geographic and economic diversity, spanning the years 1989 to 2015. Survey sites were established in both urban and rural areas. The CHNS encompasses all age groups and provides comprehensive indicators on residents’ health, nutrition, and socioeconomic conditions at the household and individual levels. It has been widely used in research related to the health of older adults and social security (29, 30).

This study focuses on the period from the late 20th century to the early 21st century, coinciding with the SOE layoffs wave. Specifically, six survey waves—1991, 1993, 1997, 2000, 2004, and 2006—are selected, consistent with prior literature (31). Given this institutional context, the 1991–2006 timeframe adopted in this study is justified. During this period, the CHNS covered the following provinces: Heilongjiang, Liaoning, Jiangsu, Shandong, Henan, Hubei, Hunan, Guangxi, and Guangdong. Considering that China’s average life expectancy in 1990 was 68.55 years,^9^ this study defines “older parents” as individuals aged 50 or older who had adult children. By matching individuals based on their “relationship to the household head,” we merge older parents with their adult children’s information, resulting in an unbalanced panel dataset.

In this study, all variables are derived from individual-level survey responses. This allows us to examine how children’s layoffs affect their parents’ health. The CHNS dataset is de-identified and publicly available for academic use. In accordance with ethical research standards and relevant data protection regulations in China and internationally, this study involves no direct contact with human subjects and uses anonymized secondary data.

### 4.2 Variables

#### 4.2.1 Independent variable

The CHNS dataset does not provide explicit information on the reasons for unemployment, but layoffs are primarily defined as job losses among employees of SOEs. Liu and Zhao (31) define laid-off workers as those who were employed in public institutions, SOEs, or collective enterprises in period *t-1* but lost their jobs by period *t*. Chen et al. (13) alternatively define laid-off samples as those who had only worked in SOEs during the survey period. Given that collective enterprises—especially small collective enterprises—were often owned by local communities (such as neighborhood offices or village cooperatives), we employ two distinct layoff definitions for robustness. Our primary definition (Layoffs Definition 1) classifies workers as laid off if they were employed at government agencies, SOEs, or large collective enterprises in *t*-*1* but lost their jobs by *t*. Accordingly, we code the dependent variable “having a laid-off child” as 1 if any adult child meets this criterion, and 0 otherwise. For sensitivity analysis, we use a broader secondary definition (Layoffs Definition 2) that additionally includes workers from small collective enterprises who experienced job loss during the same period.

#### 4.2.2 Dependent variable

This study primarily employs disease status indicators to measure the health of older adults. To achieve a more comprehensive assessment, the health measures additionally include daily functional abilities and body mass index (BMI). The disease indicators are based on self-reported illness, which generally reflects respondents’ health status adequately. However, there are two potential limitations: First, older adult respondents may not accurately perceive their health conditions—for example, reduced healthcare access due to adult children’s job loss could lead to undiagnosed illnesses. Second, some older adults may report being healthy despite having difficulties performing daily activities. The Physical Function (PF) indicator captures various constraints on daily living caused by health and physical strength limitations; however, its sample size is relatively small. BMI serves as an objective anthropometric measure. By utilizing these complementary health indicators, we can more comprehensively assess health outcomes.

First, we construct the dummy variable “Disease status” based on CHNS survey questions: “During the past 4 weeks, have you been ill or injured? Have you been diagnosed with any chronic or acute diseases?” (coded as 1 for yes, and 0 otherwise). We also examine two specific chronic conditions: hypertension and diabetes (coded as 1 if present, and 0 otherwise).

Second, to assess daily functional abilities in the older population, existing literature primarily employs three indicators: Physical Function (PF), Activities of Daily Living (ADL), and Instrumental Activities of Daily Living (IADL). Due to more severe data limitations in ADL and only four survey rounds of IADL data availability (1997–2006), this study focuses on PF measures. Following Yu and Feng (32), the PF index comprises five specific tasks: lifting heavy objects, squatting, standing up from sitting, remaining seated for 2 h, and running one kilometer. Comparatively, IADL covers more complex activities like using public transportation, cooking, shopping, telephone use, and money management. We code the variable as 0 if respondents reported “No difficulty” or “Some difficulty but still manageable” for all items, and 1 otherwise.

Finally, BMI serves as our health risk indicator, providing a comprehensive measure that reflects daily energy expenditure and intake, genetic factors, and lifestyle patterns—rather than targeting specific diseases. Using World Health Organization standards, a BMI below 18 is classified as underweight (coded as 1) and otherwise as 0. Clinically, low BMI typically results from two primary causes: inadequate nutritional intake and excessive energy expenditure. Similarly, a BMI exceeding 25 is categorized as overweight (coded as 1), and otherwise as 0.

In addition, mental health is an important dimension of older adults’ well-being. This study measures three mental health indicators. First, the mental disorder indicator is based on the survey question, “What diagnosis did the doctor give for your illness or injury?” If the respondent was diagnosed with a mental disorder, the indicator equals 1; otherwise, 0. Second, the emotional well-being indicator is derived from the question, “Are you as happy as when you were young?” Respondents who answered “strongly agree”, “agree,” or “neutral” are assigned 1; those who answered “disagree” or “strongly disagree” are assigned 0. Third, personal risk behaviors such as smoking and drinking are used as proxies for emotional stress (13). If an older adult individual smokes or drinks, the indicator is 1; otherwise, 0. Due to space constraints, the empirical results for these measures are reported in Supplementary Table S1. No significant adverse effects of adult children’s layoffs on older parents’ mental health were found in this study.

#### 4.2.3 Control variable

Consistent with Grossman’s (33) health production theory, the analysis controls for demographic characteristics (age, gender, marital status), socioeconomic factors (education), and family structure (household size). Specifically, marital status and gender are dummy variables in the empirical analysis. Education is a discrete variable, representing no education, elementary school, middle school, high school, and above.

### 4.3 Descriptive statistics

Table 1 presents the descriptive statistics of the main variables, with a total sample size of 7,332 for this study. The CHNS data provides information on respondents’ survey locations (urban areas or rural areas) and household registration status (urban hukou or rural hukou), enabling this study to examine urban–rural social security disparities. The sample is divided into three groups: older adults with urban hukou (2,490 observations, 34 percent of the sample), rural-hukou older adults living in urban areas (755 observations, 10 percent of total sample, with 80 percent of their adult children employed in SOEs, government agencies, or research institutes), and rural-hukou older adults living in rural areas (4,087 observations, 56 percent). Female respondents account for 55 percent of the sample, with the urban–rural and gender distributions being consistent with other studies. Since the sample selection requires information on both older parents’ health and their adult children’s employment, all households in the sample are necessarily multigenerational, resulting in an average household size of 4.72 persons.

**Table 1:** Descriptive statistics.

Variables	Obs.	Percent	Mean (SD)	Range
Layoffs (Definition 1)	7,332	12		0–1
Layoffs (urban hukou)	2,490	20		0–1
Layoffs (rural hukou in urban survey sites)	755	12		0–1
Layoffs (rural hukou in rural survey sites)	4,087	7		0–1
Layoffs (Definition 2)	7,332	15		0–1
Disease status (yes = 1)	7,332	15		0–1
Hypertension (yes = 1)	6,765	12		0–1
Diabetes (yes = 1)	5,163	2		0–1
PF indicator (limitation = 1)	4,241	47		0–1
IADL indicators (limitation = 1)	2,906	30		0–1
Underweight (yes = 1)	7,332	6		0–1
Overweight (yes = 1)	7,332	31		0–1
Age	7,332		62.80 (9.46)	50–101
Gender (male = 1)	7,332	45		0–1
Education	7,332		0.81 (1.07)	0–4
Marital status (in marriage = 1)	7,332	77		0–1
Hukou (urban hukou = 1)	7,332	34		0–1
Habitation (urban areas = 1)	7,332	28		0–1
Household size	7,332		4.72 (1.45)	2–13

Under Layoffs Definition 1, 12 percent of older parents had at least one adult child who experienced layoffs. The breakdown by hukou status shows: urban-hukou older parents had the highest rate (20 percent with laid-off adult children), followed by rural-hukou older parents in urban areas (12 percent with laid-off adult children), and rural-hukou older parents in rural areas (7 percent with laid-off adult children). When adopting Layoffs Definition 2 (which additionally includes workers laid off from small collective enterprises), the proportion of older parents with laid-off adult children increases to 15 percent of the total sample.

### 4.4 Estimation model

This study aims to identify the effect of adult children’s layoffs on older parents’ health. We first present baseline regression results using a panel regression model with both household and time fixed effects. We then conduct robustness checks using both the causal forest model and the logit model.

For the causal forest model, we adopt it because it yields a lower mean squared error than ordinary least squares and is better suited for capturing complex, nonlinear relationships between adult children’s layoffs and their older parents’ health. Our analysis utilizes household-level survey data, in which intricate interactions may exist among individual-level covariates (e.g., older parents education level, household size). Unlike conventional regression models that require explicit functional form assumptions, the causal forest model uses recursive partitioning and orthogonalization to flexibly estimate treatment effects. Therefore, we employ the causal forest model as a robustness check.

For the logit model, we use it to model the binary outcome in a more interpretable manner. Since the dependent variable is binary, the logit model ensures that the predicted probabilities lie within the [0, 1] interval. Although fixed-effects logit models may suffer from the parameter bias, the logit model can still serve as a robustness check to complement our primary analysis.

#### 4.4.1 Two-way fixed effects panel regression

The baseline regression model for this paper is as follows:


(1) $$H_{ih,t}=\beta_{0}+\beta_{1}layoffs_{h,t}+\beta_{2}X_{ih,t}+\nu_{t}+\mu_{h}+\varepsilon_{ih,t}$$

As shown in Equation 1, $layoffs_{h,t}$ is the explanatory variable of whether an older adult has any adult child experiencing layoff in household *h* at time *t*; $H_{ih,t}$ represents the measures of health of the older adults, including diseases status, daily behavioral ability and BMI; and $X_{ih,t}$ is the control variables. $\nu_{t}$ is a time fixed effect, $\mu_{h}$ is a household fixed effect, and $\varepsilon_{ih,t}$ is an error term.

#### 4.4.2 Causal forest model and logit model

To enhance the robustness of our findings, this study employs the causal forest model and the logit model as supplementary analytical approaches.

The causal forest method integrates random forests with partially linear regression models to develop a treatment effect estimation framework based on adaptive kernel functions (34). During data preprocessing, we strictly adhere to causal forest modeling requirements: standardizing continuous feature variables, and applying one-hot encoding to discrete variables (time periods, provinces, and households). Given the model’s sensitivity to hyperparameters, we implement grid search combined with 5-fold cross-validation for parameter optimization. This process minimizes prediction error to enhance model accuracy and ensure robust estimation results. The final hyperparameters are: 100 decision trees, maximum depth of 3, and minimum samples per node of 2. The logit model uses the same set of variables as the baseline regression to maintain comparability.

## 5 Results

### 5.1 The negative health impact of adult children’s layoffs on older parents

As shown in Table 2, adult children’s layoffs have a statistically significant negative impact on older parents’ health. Column (1) indicates that when controlling for time and province fixed effects, older parents with laid-off adult children exhibit a 2.86 percentage-point increase in the probability of disease status occurrence within the past 4 weeks. When household fixed effects are added, the coefficient of our key explanatory variable increases substantially, showing that older parents with laid-off adult children have a 4.8 percentage-point higher probability of disease status. Columns (3) and (4) present robustness checks using alternative definitions of layoffs. The baseline regression results align with theoretical predictions, confirming that adult children’s layoffs exert significant negative health shocks to older parents.

**Table 2:** The impact of adult children’s layoffs on older parents’ health.

Variables	Layoffs (Definition 1)		Layoffs (Definition 2)	
	Disease status	Disease status	Disease status	Disease status
	(1)	(2)	(3)	(4)
Layoffs	0.0286**	0.048**	0.0207*	0.0331*
	(0.0137)	(0.0243)	(0.0121)	(0.0195)
Age	0.0026***	0.0042***	0.0026***	0.0041***
	(0.0005)	(0.0016)	(0.0005)	(0.0016)
Gender	−0.0153*	−0.0145	−0.0141	−0.0141
	(0.0089)	(0.0102)	(0.0089)	(0.0102)
Education (primary)	0.0169	0.0179	0.0173	0.0180
	(0.0108)	(0.0158)	(0.0108)	(0.0158)
Education	0.0032	0.0079	0.0036	0.0096
(lower secondary)	(0.0137)	(0.0198)	(0.0137)	(0.0198)
Education	0.0423*	0.0554	0.0413*	0.0558
(upper secondary)	(0.0229)	(0.0350)	(0.0228)	(0.0349)
Education (above)	0.00524	0.0189	0.00499	0.0201
	(0.0246)	(0.0385)	(0.0245)	(0.0385)
Marital status	−0.0239*	0.0201	−0.0244**	0.0188
	(0.0124)	(0.0280)	(0.0124)	(0.0279)
Household size	0.0018	0.0044	0.0016	0.0043
	(0.0029)	(0.0061)	(0.0029)	(0.0061)
Time fixed effect	Yes	Yes	Yes	Yes
Province fixed effects	Yes	No	Yes	No
Household fixed effect	No	Yes	No	Yes
Observations	7,332	6,900	7,332	6,900
*R*-squared	0.049	0.384	0.048	0.383

***, ** and * indicate significance at the 1, 5 and 10% levels, respectively. Standard error clustering at the household level.

### 5.2 Heterogeneous effects of urban–rural social security

For older adults with access to social security, the negative health impacts of adult children’s layoffs are mitigated. However, during the study period, rural-hukou older parents residing in urban areas—who lacked adequate social protection—suffered stronger adverse health effects when their adult children experienced job loss. As shown in Table 3, we conduct subgroup analyses by dividing the sample into four categories: Column (1) presents results for the full sample; Column (2) for rural-hukou older parents in urban areas; Column (3) for urban-hukou older parents in urban areas; and Column (4) for rural-hukou older parents in rural areas. The results reveal that children’s layoffs primarily negatively affected rural-hukou older parents living in urban areas, increasing their probability of reporting illness by 14 percentage points and hypertension prevalence by 11 percentage points. The inconsistent results for diabetes (compared to hypertension) may stem from different etiologies—while hypertension can be exacerbated by psychological stress (e.g., anxiety from children’s unemployment), diabetes is more closely associated with dietary and lifestyle factors.

**Table 3:** Heterogeneous effects of urban–rural social security.

Variables	(1) Full sample	(2) Rural hukou; Urban areas	(3) Urban hukou	(4) Rural hukou; Rural areas
Disease status				
Disease status	0.048**	0.1360**	0.0363	0.00933
	(0.0243)	(0.0659)	(0.0385)	(0.0344)
Observations	6,900	671	2,168	3,860
Hypertension	0.0085	0.1120*	0.0265	−0.0292
	(0.0173)	(0.0584)	(0.0248)	(0.0221)
Observations	6,349	604	1,988	3,530
Diabetes	0.0110	0.0009	0.0245	0.0007
	(0.0113)	(0.0018)	(0.0167)	(0.0234)
Observations	4,786	461	1,438	2,695
Activities of daily living				
PF	0.0543	0.3030*	0.0456	−0.0383
	(0.0415)	(0.1790)	(0.0528)	(0.0871)
Observations	3,811	299	1,370	1,976
IADL	0.0522	−0.1090	0.0597	0.0103
	(0.0429)	(0.1810)	(0.0543)	(0.1450)
Observations	2,503	216	911	1,255
BMI				
Underweight	−0.0052	0.0706*	−0.0001	−0.0221
	(0.0104)	(0.0413)	(0.0157)	(0.0190)
Observations	6,967	674	2,188	3,872
Overweight	−0.0181	−0.0076	0.0064	−0.0346
	(0.0220)	(0.0684)	(0.0330)	(0.0363)
Observations	6,967	674	2,188	3,872
Control variable	Yes	Yes	Yes	Yes
Time fixed effect	Yes	Yes	Yes	Yes
Household fixed effect	Yes	Yes	Yes	Yes

***, ** and * indicate significance at the 1, 5 and 10% levels, respectively. Standard error clustering at the household level. All regression control variables in this paper include age, gender, education, marital status, and household size.

Furthermore, adult children’s layoffs significantly increased PF limitations only among rural-hukou older parents living in urban areas, with no observable effects on other groups. Regarding BMI, rural-hukou older parents living in urban areas showed higher probabilities of being underweight (likely due to reduced nutritional intake after adult children’s job loss), but no significant changes in overweight prevalence. These findings confirm Hypothesis 2: under China’s urban–rural social security divide, rural-hukou older parents in urban areas were most vulnerable to the adverse health impacts of adult children’s layoffs.

### 5.3 Robustness tests—causal forest model and logit model results

Supplementary Table S2 presents the estimation results from both the causal forest model and Logit model, examining the impact of adult children’s layoffs on older parents’ disease status. Columns (1) and (2) report causal forest model estimates. Similar to fixed-effects models, these employ province-level and household-level clustering algorithms, respectively, to control for unobserved, time-invariant characteristics at either the provincial or household level. When using household-level clustering, the estimated average treatment effect (ATE) of having a laid-off child on older parents’ disease probability is 0.031. Columns (3) and (4) display Logit regression results incorporating province fixed effects and household fixed effects, respectively. The findings remain robust across these alternative specifications, consistently demonstrating significant negative health consequences.

### 5.4 Mechanism testing

This study posits that layoffs significantly reduce household income, thereby diminishing financial support for older parents and ultimately impairing their health. To elucidate this pathway, we empirically examine how adult children’s job loss affects household income levels, older parents’ nutritional intake, healthcare utilization, daily care provision.

#### 5.4.1 Household income

Income serves as a fundamental determinant of health outcomes, with socioeconomic disparities in income and living circumstances driving persistent health inequalities (35). For laid-off workers from SOEs, re-employment proves particularly challenging. Consequently, the decline in household income resulting from adult children’s layoffs may adversely affect older parents’ health. As shown in Table 4, after controlling for both time and household fixed effects, families with adult children’s layoffs suffer a statistically significant 34 percent reduction in income compared to those without such job losses. This negative impact remains robust when additional control variables are incorporated, although the coefficient undergoes minor adjustments. These findings consistently demonstrate that adult children’s layoffs exert a stable negative effect on household income.

**Table 4:** The impact of adult children’s layoffs on household income.

Variables	Household income	Household income
	(1)	(2)
Layoffs	−0.3432***	−0.3215***
	(0.0879)	(0.1030)
Control variable	No	Yes
Time fixed effect	Yes	Yes
Household fixed effect	Yes	Yes
Observations	6,900	6,900
R-squared	0.618	0.634

The household income indicator takes the value of ln (net household income/CPI+1). ***, ** and * indicate significance at the 1, 5 and 10% levels, respectively. Standard error clustering at the household level.

#### 5.4.2 Nutritional intake

The intake of nutrients significantly impacts the health status of older adults, while “income shocks” can alter household dietary structures (28). When adult children experience layoffs, older parents may face tighter food consumption constraints, leading to reduced nutrient intake and consequent health deterioration. Due to disparities in access to social security, this negative effect is most severe among rural-hukou older parents residing in urban areas.

As shown in Table 5, layoffs among adult children significantly reduce the intake of calories, proteins, and fats among rural-hukou older parents in urban areas, whereas no such effect is observed for urban-hukou older parents or rural residents. The results for carbohydrate intake are statistically insignificant, likely because staple foods like rice and wheat flour are relatively affordable—while layoffs increase food consumption constraints for the older parents, they may not substantially restrict the consumption of inexpensive carbohydrates.

**Table 5:** The impact of adult children’s layoffs on nutritional intake.

Variables	(1) Full sample	(2) Rural hukou; Urban areas	(3) Urban hukou	(4) Rural hukou; Rural areas
Calories	−0.0387	−0.107*	−0.0291	−0.0268
	(0.0236)	(0.0606)	(0.0344)	(0.0437)
Observations	6,703	653	2,095	3,732
Proteins	−0.0261	−0.1309**	−0.0071	−0.0044
	(0.0259)	(0.0582)	(0.0388)	(0.0446)
Observations	6,703	653	2,095	3,732
Fats	−0.0492	−0.213*	−0.008	−0.0065
	(0.0388)	(0.109)	(0.0583)	(0.0626)
Observations	6,703	653	2,095	3,732
Carbohydrate	−0.029	−0.0888	−0.0228	−0.0305
	(0.0257)	(0.0662)	(0.0370)	(0.0469)
Observations	6,703	653	2,095	3,732
Control variable	Yes	Yes	Yes	Yes
Time fixed effect	Yes	Yes	Yes	Yes
Household fixed effect	Yes	Yes	Yes	Yes

The explanatory variables are the logarithmic forms of three-day average caloric intake (kcal), three-day average protein intake (g), three-day average fat intake (g), and three-day average carbohydrate intake (g), respectively. ***, ** and * indicate significance at the 1, 5 and 10% levels, respectively. Standard error clustering at the household level.

#### 5.4.3 Healthcare utilization

The extent of healthcare utilization directly impacts the health status of older adults, with factors such as the number of working family members and disposable income serving as key determinants of individual healthcare utilization (36). When adult children experience job loss, older parents may demonstrate reduced healthcare utilization. We employ two measures from the CHNS database: healthcare access (a dummy variable based on “Have you visited a formal medical institution in the past 4 weeks?”, where 1 indicates yes and 0 indicates no), and medical expenditures (total treatment costs minus the portion covered by medical insurance, plus any additional out-of-pocket expenses for treatment). Due to data limitations, both indicators have small sample sizes: the healthcare access data were only available for 2004 and 2006, while the medical expenditure data were only reported by respondents who were ill.

As shown in Table 6, while no significant reduction in the probability of seeking medical care was observed among older parents with adult children’s layoffs, the medical expenditures of ill older parents with such children were significantly lower than those of other ill older parents. This provides suggestive evidence that adult children’s layoffs negatively affect the healthcare utilization of older parents, leading to a phenomenon of foregone necessary medical care.

**Table 6:** The impact of adult children’s layoffs on healthcare utilization and daily care.

Variables	Healthcare access	Medical expenditures	Cleaning	Cooking	Laundry	Buying food
Layoffs	0.0036	−0.7905*	−0.1952	0.1125	−0.2901	0.0262
	(0.0052)	(0.439)	(0.2081)	(0.1263)	(0.2124)	(0.4543)
Control variable	Yes	Yes	Yes	Yes	Yes	Yes
Time fixed effect	Yes	Yes	Yes	Yes	Yes	Yes
Household fixed effect	Yes	Yes	Yes	Yes	Yes	Yes
Observations	1,446	303	998	1,025	1,660	910
R-squared	0.529	0.520	0.820	0.810	0.761	0.787

The medical expenditure indicator takes the value of ln (medical expenditure/CPI+1). ***, ** and * indicate significance at the 1, 5 and 10% levels, respectively. Standard error clustering at the household level.

#### 5.4.4 Daily care

Displaced workers experienced significant reductions in employment and work hours (12). Therefore, layoffs can alter adult children’s income and leisure time, thereby affecting their daily caregiving for older parents. The CHNS database provides data on respondents’ average daily time spent on cleaning, laundry, grocery shopping, and cooking for their families. This study focuses on multi-generational co-resident households, where such housework time is regarded as adult children’s daily care for older parents. To account for varying numbers of children per older parent, we calculate the mean time each child spends on domestic tasks, then use the logarithmic value as a proxy for daily care received. Regression results in Table 6 show no significant change in average daily care time provided by laid-off adult children, indicating that deteriorating health among older adults in affected households cannot be attributed to reduced daily care by their children.

To further explore the impact of adult children’s layoffs on daily care, we separately test the effects of sons’ and daughters’ layoffs on caregiving time. Supplementary Table S3 show, no significant impact from either. We also examine caregiving quality using adult children’s smoking and drinking as proxies. As shown in Supplementary Table S4, layoffs do not lead to increased smoking or drinking among adult children, suggesting no deterioration in caregiving quality.

## 6 Discussion

The results indicate that layoffs significantly increase the risk of morbidity among older parents. This effect is particularly pronounced among rural-hukou older parents residing in urban areas. Specifically, adult children’s layoffs lead to higher rates of illness, an increased likelihood of hypertension, greater limitations in activities of daily living, and a higher probability of underweight status within this subgroup. Mechanism analysis reveals that the adverse health effects are not primarily driven by reduced caregiving from adult children, but rather by household income loss. The resulting income shock diminishes older parents’ nutritional intake—especially protein and fat consumption—and impairs their healthcare utilization.

The disproportionate impact of adult children’s layoffs on rural older parents living in urban areas highlights both the disparities in urban–rural social security systems and the role of the social safety net. It also underscores the vulnerability of mobile older adults, who suffer a “double burden” of inadequate social protection and the high cost of living in cities. On the one hand, during the wave of layoffs, the highly segmented urban–rural social security system left rural hukou holders without access to basic welfare benefits. Migrant rural older adults often face institutional barriers in obtaining urban household registration (hukou), which restricts their access to urban social security programs (26, 27). On the other hand, living in urban areas imposes higher living expenses. Unlike in rural areas, where subsistence farming can offset food costs, these individuals must rely more heavily on market-based services.

In recent years, labor market structures and social security systems have undergone significant transformation. Nevertheless, the findings of this study remain highly relevant in the context of rising unemployment risks and population aging. First, unemployment risks have been exacerbated by trade tensions and rapid technological advancement. Heightened trade frictions have increased global uncertainty, and aggressive tariff policies may lead to surging unemployment rates. Both the International Monetary Fund and the World Bank have emphasized the urgency of resolving trade disputes to prevent the spread of systemic risks. Meanwhile, the rapid development of robotics and artificial intelligence has raised the risk of structural unemployment and declining labor income (37, 38). Second, although China has significantly expanded the coverage and improved the level of its social security system, notable gaps remain. As of now, over 95% of the population is covered by basic medical insurance. However, the Urban–Rural Resident Basic Medical Insurance still suffers from high deductibles, low reimbursement rates, and insufficient caps, leaving many with a considerable healthcare burden (7). Regarding pensions, in 2025, the minimum monthly basic pension for urban and rural residents stands at just 143 yuan, which falls far short of covering average living expenses. Therefore, this study reinforces the urgency of strengthening social security systems to safeguard vulnerable households in times of rising global unemployment risks.

This study makes three contributions. First, it provides a theoretical contribution by highlighting the protective role of social security systems in mitigating intergenerational welfare shocks. Existing literature has paid limited attention to the role of social security in safeguarding older parents’ health when adult children experience unemployment. This study addresses this gap by exploring the relationship between social protection and older adults’ health in the context of labor market frictions. Second, it provides novel empirical evidence on how macroeconomic shocks transmit into micro-level welfare losses, and offers policy insights by recommending the gradual and socially sensitive implementation of structural reforms. Third, by leveraging SOE reforms as exogenous policy shocks, this study further isolates the causal effect of adult children’s unemployment on older parents’ health, thereby enhancing the credibility of the findings.

This study also has several limitations. First, while the CHNS provides rich longitudinal data, it does not include reliable mental health indicators for the sample period analyzed. As a result, this study does not observe significant adverse impact of adult children’s layoffs on older parents’ psychological well-being. Second, the study is grounded in the Chinese cultural context, where family-based older adult care remains predominant. This cultural specificity may limit the generalizability of the findings to other societies with weaker intergenerational support. Future research could explore whether similar intergenerational health impacts exist in such contexts, particularly with respect to mental health.

## 7 Conclusion

Against the dual challenges of population aging and labor market restructuring, this study investigates the health risks faced by households where adult children are unemployed and the protective role of social security systems, offering important policy implications. This study utilizes China’s SOE reforms as a quasi-natural experiment to examine the adverse health effects of adult children’s unemployment on older parents’ health, with particular emphasis on establishing causal relationships. Using data from the CHNS (1991–2006), we examine how adult children’s layoffs during the SOE reforms affect older parents’ health outcomes and the underlying mechanisms, with particular focus on disparities caused by urban–rural differences in social security coverage. Employing two-way fixed effects models and causal forest methods, the analysis yields three key findings. First, adult children’s layoffs significantly worsened older parents’ health status. Second, due to urban–rural disparities in social security, this negative effect was most pronounced among rural-hukou older parents living in urban areas, manifesting through elevated disease status, limitations in daily activities, and higher probability of being underweight. Third, the mechanism operated through reduced household income, which decreased older parents’ protein and fat intake and healthcare utilization—effects that were particularly severe for rural-hukou older parents in urban areas.

The findings of this study indicate that macro-level policies, such as SOE reforms, can lead to significant welfare losses for households. As the government rapidly adjusts economic and trade policies, it must also prioritize strengthening the social security system to mitigate the adverse impacts on unemployed families.

## Data Availability

Publicly available datasets were analyzed in this study. This data can be found at: https://www.cpc.unc.edu/projects/china/data.

## Footnotes

^1^Available online at: https://cn.weforum.org/publications/the-future-of-jobs-report-2025/digest/ (accessed April 20, 2025).

^2^Data from the 2025 State Council Government Work Report. Available online at: https://www.gov.cn/yaowen/liebiao/202503/content_7013163.htm?s_channel=5&s_trans=7824452999_ (accessed April 20, 2025).

^3^National Bureau of Statistics: Statistical Bulletin of the People’s Republic of China on National Economic and Social Development in 2024. Available online at: https://www.gov.cn/lianbo/bumen/202502/content_7008605.htm (accessed April 20, 2025).

^4^Available online at: https://www.stats.gov.cn/sj/pcsj/rkpc/7rp/indexch.htm (accessed April 20, 2025).

^5^Data from the China Statistical Yearbook.

^6^Data from the 1998 State Council Government Work Report. Available online at: https://www.gov.cn/test/2006-02/16/content_201129.htm (accessed April 20, 2025).

^7^Available online at: https://www.gov.cn/gongbao/content/2001/content_60684.htm (accessed April 20, 2025).

^8^Available online at: https://www.gov.cn/zhengce/zhengceku/2009-09/04/content_7280.htm (accessed April 20, 2025).

^9^Data from National Bureau of Statistics. Available online at: https://data.stats.gov.cn/easyquery.htm?cn=C01&zb=A0304&sj=2020 (accessed April 20, 2025).

## References

[1] Hilger NG. Parental job loss and children's long-term outcomes: evidence from 7 million fathers' layoffs. Am Econ J Appl Econ. (2016) 8:247–83. doi: 10.1257/app.20150295

[2] Xie W. Generations at the crossroads: biographical experience and working-class politics in China. Labor Hist. (2024) 65:337–51. doi: 10.1080/0023656x.2024.2319298

[3] Song Q Lim E Friedman E Smith JP. Impact of layoffs on mortality and physical health in transitional China 1989–2015. Soc Sci Res. (2025) 125:103110. doi: 10.1016/j.ssresearch.2024.103110, PMID: 39615962

[4] Jiang Z Liu H Deng J Ye Y Li D. Influence of intergenerational support on the mental health of older people in China. PLoS One. (2024) 19:e0299986. doi: 10.1371/journal.pone.0299986, PMID: 38635847 PMC11025908

[5] Huo M Graham JL Kim K Birditt KS Fingerman KL. Aging parents’ daily support exchanges with adult children suffering problems. J Gerontol: Series B. (2019) 74:449–59. doi: 10.1093/geronb/gbx079, PMID: 28633505 PMC6377028

[6] Zhao Y. Soe layoffs, family resources and children's education (in Chinese). Econ Res J. (2016) 51:101–15.

[7] Liu C Kong Y Su Q Xing H Tian Z. The impact of the urban–rural residents’ medical insurance integration on rural residents’ out-of-pocket medical costs: based on the deductible, reimbursement rate, and ceiling line. Front Public Health. (2025) 13:1576978. doi: 10.3389/fpubh.2025.1576978, PMID: 40265068 PMC12011745

[8] Yu W Gao Y Wang R Feng X Sun R Huang Y. Olg model analysis of delayed retirement and social pension effects on family-based elderly care in China. Mathematics. (2024) 12:3314. doi: 10.3390/math12213314

[9] Xia M. The Soe reform, massive layoff and private economy boom in China. Kyoto Econ Rev. (2025) 198:85–119. doi: 10.57475/keizaironso.198.1.5

[10] Bai C-E Lu J Tao Z. The multitask theory of state enterprise reform: empirical evidence from China. Am Econ Rev. (2006) 96:353–7. doi: 10.1257/000282806777212125

[11] NBS. The scale of employment continues to expand, and the employment situation remains stable in the long run (in Chinese). China Information News. (2019)

[12] Tian X Gong J Zhai Z. The effect of job displacement on labor market outcomes: evidence from the Chinese state-owned enterprise reform. China Econ Rev. (2022) 72:101743. doi: 10.1016/j.chieco.2022.101743

[13] Chen Q Hu Y Fu H. The short-and long-term impacts of unemployment on health: evidence from massive layoff of Chinese Soes' Workers in the late 1990s (in Chinese). Chin J Popul Sci. (2017) 31:51–61.

[14] Kong N Osberg L Zhou W. The shattered “Iron Rice bowl”: intergenerational effects of Chinese state-owned Enterprise reform. J Health Econ. (2019) 67:102220. doi: 10.1016/j.jhealeco.2019.06.007, PMID: 31330471

[15] Wu L Xie T Guan W Li W. Impact of intergenerational support on older adults’ care expectations in rural areas in China. Front Public Health. (2024) 12:1423173. doi: 10.3389/fpubh.2024.1423173, PMID: 39575105 PMC11579706

[16] Zhang P Liu Y. The higher the children's achievements, the better the elderly health? Evidence from China. Front Public Health. (2022) 10:871266. doi: 10.3389/fpubh.2022.871266, PMID: 35719647 PMC9204310

[17] Yang Y Evandrou M Vlachantoni A. The impact of living arrangements and intergenerational support on the health status of older people in China: are rural residents disadvantaged compared to urban residents? Ageing Soc. (2023) 43:469–94. doi: 10.1017/s0144686x21000702

[18] Bridges S Liu L. The effect living arrangements and intergenerational support have on the incidence of catastrophic health expenditure: a microeconomic analysis for China. China Econ Rev. (2025) 90:102360. doi: 10.1016/j.chieco.2025.102360

[19] Torssander J. Adult children's socioeconomic positions and their parents' mortality: a comparison of education, occupational class, and income. Soc Sci Med. (2014) 122:148–56. doi: 10.1016/j.socscimed.2014.10.043, PMID: 25441327

[20] Choi EJ Choi J Son H. The long-term effects of labor market entry in a recession: evidence from the Asian financial crisis. Labour Econ. (2020) 67:101926. doi: 10.17848/wp19-312 32989344 PMC7510546

[21] Haapanen M Pehkonen J Seppälä V. Parental earnings response to children's job loss: evidence from Finland. Labour Econ. (2025) 94:102714. doi: 10.2139/ssrn.4963016

[22] McGarry K. Dynamic aspects of family transfers. J Public Econ. (2016) 137:1–13. doi: 10.1016/j.jpubeco.2016.03.008

[23] Zuo T Jin J Alcock R. China’s social welfare system in transition In: Carr H, Kirton-Darling E, Meers J, Salcedo Repolês MF, editors. Research Handbook on Social Welfare Law. Cheltenham: Edward Elgar Publishing (2024). 171–88.

[24] Wang L Liu J. The impact of long-term care insurance on family Care for Older Adults: the mediating role of intergenerational financial support. PLoS One. (2024) 19:e0299974. doi: 10.1371/journal.pone.0299974, PMID: 38781177 PMC11115205

[25] Wang Z Li X Chen M Si L. Social health insurance, healthcare utilization, and costs in middle-aged and elderly community-dwelling adults in China. Int J Equity Health. (2018) 17:1–13. doi: 10.1186/s12939-018-0733-0 29394933 PMC5797397

[26] Ma C Song Z Zong Q. Urban-rural inequality of opportunity in health care: evidence from China. Int J Environ Res Public Health. (2021) 18:7792. doi: 10.3390/ijerph18157792, PMID: 34360084 PMC8345655

[27] Cui Y Yang F. Accessibility of basic public health service promotes social integration of elderly migrants in China. Sci Rep. (2025) 15:10685. doi: 10.1038/s41598-025-95146-z, PMID: 40155687 PMC11953308

[28] Wang G Hao Y Ma J. Family income level, income structure, and dietary imbalance of elderly households in rural China. Foods. (2024) 13:190. doi: 10.3390/foods13020190, PMID: 38254491 PMC10814872

[29] Luo L Zeng X Wang X. The effects of health insurance and physical exercise participation on life satisfaction of older people in China—based on Chns panel data from 2006 to 2015. Front Public Health. (2022) 10:858191. doi: 10.3389/fpubh.2022.858191, PMID: 36091561 PMC9458912

[30] Fu W Zhu F Cheng Y. Gender differences in intergenerational effects of laid-off parents. Econ Syst. (2023) 47:101120. doi: 10.1016/j.ecosys.2023.101120

[31] Liu H Zhao Z. Parental job loss and children's health: ten years after the massive layoff of the Soes' Workers in China. China Econ Rev. (2014) 31:303–19. doi: 10.1016/j.chieco.2014.10.007

[32] Yu Y Feng J. The dynamic changes in elderly health in China and their implications for healthy aging (in Chinese). World Econ Papers. (2017) 1:1–16.

[33] Grossman M. On the concept of health capital and the demand for health. Determinants of health: An economic perspective. New York: Columbia University Press (2017). p. 6–41.

[34] Wager S Athey S. Estimation and inference of heterogeneous treatment effects using random forests. J Am Stat Assoc. (2018) 113:1228–42. doi: 10.1080/01621459.2017.1319839

[35] Ozkan S. Income differences and health disparities: roles of preventive vs. curative medicine. J Monet Econ. (2025) 150:103698. doi: 10.1016/j.jmoneco.2024.103698

[36] Özkaya MH Alakbarov N Gündüz M. The relationship between health-care expenditure and disposable personal income: a panel econometric analysis on the Eu countries. Int J Hum Rights Healthc. (2024) 17:736–50. doi: 10.1108/ijhrh-04-2021-0103

[37] Acemoglu D. The simple macroeconomics of AI. Econ Policy. (2025) 40:13–58. doi: 10.1093/epolic/eiae042

[38] Wang L Zhou H Wan G. The impact of robots on unemployment duration: evidence from the Chinese general social survey. China Econ Rev. (2025) 89:102305. doi: 10.1016/j.chieco.2024.102305

