# Supplementary material for: Urban–rural social security, adult children’s layoffs and older parents’ health
Source: Front Public Health. 2025 Sep 3;13:1619806. doi: 10.3389/fpubh.2025.1619806 (PMC12440936; doi:10.3389/fpubh.2025.1619806)
Supplement: Supplementary file 1 [file Table_1.docx]

Supplementary Material

# The impact of adult children's layoffs on mental health

As shown in Supplementary Table S1, no significant negative effects of adult children’s layoffs on the mental health of older parents were found in this study. Specifically, Column (1) indicates that adult children’s layoffs have no significant impact on the likelihood of older parents suffering from mental disorders; Column (2) shows no significant effect on older parents’ emotional well-being (whether they feel happy); Columns (3) and (4) reveal that layoffs do not significantly influence older parents’ engagement in risk behaviors such as alcohol consumption and smoking.

It is important to note that although our results do not indicate significant adverse effects of adult children’s layoffs on older parents’ mental health, this finding may be limited by the historical context of the study period and the relatively low public awareness of mental health issues at that time.

*[S1 Table]*

# Tables

S1 Table. The impact of adult children's layoffs on mental health

| Variables | Mental illness | Affective indicators | Drinking | Smoking |
| --- | --- | --- | --- | --- |
| Layoffs | -0.0011 | 0.1976 | 0.0198 | -0.0285 |
|  | (0.0021) | (0.1793) | (0.0222) | (0.0178) |
| Control variable | Yes | Yes | Yes | Yes |
| Time fixed effect | Yes | No | Yes | Yes |
| Household fixed effect | Yes | No | Yes | Yes |
| Observations | 1453 | 717 | 6,459 | 6,508 |
| R-squared | 0.840 | 0.095 | 0.585 | 0.641 |

Note: Affective indicators are only available for the 2006 wave, which prevents the inclusion of fixed effects in the analysis.

S2 Table. Robustness tests - causal forest model and logit model results

|  | Causal forest model | | Logit model | |
| --- | --- | --- | --- | --- |
| Variables | Disease status | Disease status | Disease status | Disease status |
|  | (1) | (2) | (3) | (4) |
| Layoffs | 0.0316*** | 0.031*** | 0.2556** | 0.3722** |
|  | (0.0012) | (0.0012) | (0.0997) | (0.1882) |
| Time fixed effects | Yes | Yes | Yes | Yes |
| Province fixed effects | Yes | No | Yes | No |
| Household fixed effects | No | Yes | No | Yes |
| Observations | 7,332 | 7,332 | 7,332 | 2,894 |

S3 Table. The impact of adult children's layoffs on daily care (by gender of the adult child)

| Variables | Cleaning | | Cooking | | Laundry | | Buying food | |
| --- | --- | --- | --- | --- | --- | --- | --- | --- |
| Son's layoffs | -0.3678 |  | 0.0296 |  | -0.3196 |  | 0.6865 |  |
|  | (0.2144) |  | (0.1170) |  | (0.2354) |  | (0.5590) |  |
| Daughter’s |  | -0.1108 |  | 0.0536 |  | -0.3972 |  | -0.4162 |
| layoffs |  | (0.2694) |  | (0.1534) |  | (0.2895) |  | (0.5473) |
| Control variable | Yes | Yes | Yes | Yes | Yes | Yes | Yes | Yes |
| Time fixed effect | Yes | Yes | Yes | Yes | Yes | Yes | Yes | Yes |
| Household fixed effect | Yes | Yes | Yes | Yes | Yes | Yes | Yes | Yes |
| Observations | 998 | 998 | 1,025 | 1,025 | 1,660 | 1,660 | 910 | 910 |

S4 Table. The impact of adult children's layoffs on the quality of care

| Variables | Adult children's smoking | Adult children's smoking |
| --- | --- | --- |
| Layoffs | -0.0616 | 0.0438 |
|  | (0.0418) | (0.0332) |
| Control variable | Yes | Yes |
| Time fixed effect | Yes | Yes |
| Household fixed effect | Yes | Yes |
| Observations | 7,732 | 7,732 |
| R-squared | 0.576 | 0.629 |
